# Effect of Feeding Palm Oil By-Products Based Diets on Total Bacteria, Cellulolytic Bacteria and Methanogenic Archaea in the Rumen of Goats

**DOI:** 10.1371/journal.pone.0095713

**Published:** 2014-04-22

**Authors:** Abdelrahim Abubakr, Abdul Razak Alimon, Halimatun Yaakub, Norhani Abdullah, Michael Ivan

**Affiliations:** 1 Department of Animal Science, University Putra Malaysia, Serdang, Selangor, Malaysia; 2 Institute of Tropical Agriculture, University Putra Malaysia, Serdang, Selangor, Malaysia; 3 Department of Biochemistry, University Putra Malaysia, Serdang, Selangor, Malaysia; 4 Department of Animal Nutrition, Faculty of Animal production, University of Bahri, Khartoum, Sudan; Auburn University, United States of America

## Abstract

Rumen microorganisms are responsible for digestion and utilization of dietary feeds by host ruminants. Unconventional feed resources could be used as alternatives in tropical areas where feed resources are insufficient in terms of quality and quantity. The objective of the present experiment was to evaluate the effect of diets based on palm oil (PO), decanter cake (DC) or palm kernel cake (PKC) on rumen total bacteria, selected cellulolytic bacteria, and methanogenic archaea. Four diets: control diet (CD), decanter cake diet (DCD), palm kernel cake diet (PKCD) and CD plus 5% PO diet (CPOD) were fed to rumen cannulated goats and rumen samples were collected at the start of the experimental diets (day 0) and on days 4, 6, 8, 12, 18, 24 and 30 post dietary treatments. Feeding DCD and PKCD resulted in significantly higher (P<0.05) DNA copy number of total bacteria, *Fibrobacter succinogenes*, *Ruminococcus flavefeciens*, and *Ruminococcus albus*. Rumen methanogenic archaea was significantly lower (P<0.05) in goats fed PKCD and CPOD and the trend showed a severe reduction on days 4 and 6 post experimental diets. In conclusion, results indicated that feeding DCD and PKC increased the populations of cellulolytic bacteria and decreased the density of methanogenic archaea in the rumen of goats.

## Introduction

Limited feed resources are the most important constraint for animal production industries in tropical areas. Agro-industrial by-products could be alternative as cheap and sustainable feed for ruminants. Oil palm industry in Malaysia produces annually huge amounts of biomass available as potential animal feed, such as oil palm frond, palm oil (PO), decanter cake (DC) and palm kernel cake (PKC). However, utilization of these by-products by animals is highly dependent on the rumen microbial activity to produce short chain fatty acids and microbial protein. Many studies reported that feeding PKC to cattle [1], sheep [2] and goats [3] resulted in reduced microorganisms in the rumen. However, the activities of bacteria (especially cellulolytic bacteria) in the rumen of the goats fed oil palm by-products are yet unknown. Bacteria are the most numerous microorganisms in the rumen and play a major role in the degradation of dietary fiber [4]. *Fibrobacter succinogenes*, *Ruminococcus albus*, and *Ruminococcus flavefaciens* are presently recognized as the major cellulolytic bacterial species found in the rumen [5], [6]. The presence or absence of rumen ciliate protozoa is reported to be associated with changes in the composition of the bacterial population [7].

Traditional methods of studying rumen microorganisms such as role-tube techniques [8] or most probable-number estimates [9] are found to be laborious and time consuming. Moreover, some rumen microorganisms cannot be cultivated with current techniques, whilst the cultured microorganisms represent only small fraction of the natural microbial population, hence the microbial diversity is grossly under estimated [10], [11]. Recent advances in molecular biology techniques allow the analysis of such bacteria without culturing. The development of real time polymerase chain reaction (real-time PCR) has been successfully used to quantify rumen protozoa [12]–[13] cellulolytic fungi [14] and cellulolytic bacteria species [4]–[15]. Reliability and simplicity are the advantages of this technique. Feed intake, nutrient digestibility and rumen fermentation characteristics in the goats fed palm oil by-products have been detailed in our previous report [16]. To the best of our knowledge, information on the population of microorganisms in the rumen of goats fed palm oil by-products is very limited. Therefore, the objective of the present study was to determine the effect of feeding DC, PKC and PO on the cellulolytic bacteria and methanogenic archaea in the rumen of goats.

## Materials and Methods

### Animals, diets and sample collection

The experimental protocol was approved by the University Putra Malaysia Animal Care and Use Committee, and care of the experimental goats was in accordance with the Malaysian standards.

Experimental details on animals, diets and sampling protocol were published elsewhere [16]. Briefly, 16 cannulated cross breed goats with initial live weight of 28±4 kg housed in individual pens were used in the present experiment. Four goats were assigned according to total rumen protozoa counts to one of the following dietary concentrate treatments; control diet (CD), decanter cake diet (DCD), palm kernel cake diet (PKCD), and CD plus 5% PO diet (CPOD) where PO replaced an equal amount of corn grain in CD. The concentrate diets were fed in limited amounts (1.5% body weight) once daily at 0800 h; to minimize refusals and differences in intakes among the dietary treatments. In addition, rice straw was available to all goats *ad libitum* during the entire experiment. All groups were fed CD for 28 days (pre-experiment days -28 to -1) and then fed one of the respective dietary treatments for 30 days (days 0 to 30). The experiment lasted for 58 days and the rumen digesta samples were collected during the last 30 days. Experimental concentrate mixtures were formulated to provide approximately equal amounts of crude protein (CP, 15%) and oil (6%) on dry matter (DM) basis. Chemical composition and ingredients of experimental diets are summarized in **Table 1**. Fresh water and salt blocks were available *ad libitum* throughout the experiment.

**Table 1:** Ingredients (%) and their chemical composition (% DM) of decanter cake, palm kernel cake and experimental diets.

Ingredients/Diets	PO	PKC	DC	CD	DCD	PKCD	CPOD
Rice straw				10	10	10	10
Corn grain				65	1	1	60
Soybean meal				22	6	6	22
Palm kernel cake				0	0	80	0
Decanter cake				0	80	0	0
Palm oil				0	0	0	5
Molasses				1.25	1.25	1.25	1.25
Urea				0.75	0.75	0.75	0.75
Salt				0.5	0.5	0.5	0.5
Dicalcium phosphate				0.5	0.5	0.5	0.5
Chemical composition							
Dry matter %		94.7	72.4	89.5	75.9	93.8	95.9
Organic matter		93.9	93.7	95.7	91.5	93.3	96.0
Crude protein		15.9	15.7	16.4	16.3	16.3	16.0
Ether extract		9.1	10.9	2.8	7.4	7.6	7.6
Ash		6.1	6.3	4.3	8.5	6.7	4.0
Neutral detergent fiber		72.3	45.6	19.7	45.6	67.0	19.1
Acid detergent fiber		47.6	17.2	10.2	19.8	44.1	10.0
Acid detergent lignin		17.3	13.8	1.1	6.1	14.5	1.1
Fatty acid composition (g/100 g fatty acids)							
C12:0	1.69	52.13	0.47	NA	0.02	53.41	0.01
C14:0	0.58	15.38	0.88	NA	0.50	16.21	0.81
C16:0	49.64	8.65	38.98	9.87	40.35	4.26	45.53
C18:0	3.76	4.63	4.65	5.82	5.80	11.21	4.45
C18:1	35.4	15.64	43.77	29.43	38.76	7.80	40.64
C18:2	7.34	1.39	9.44	54.43	12.12	4.57	8.94
C18:3	1.08	1.77	1.38	0.51	2.34	2.44	0.22
C20:0	0.51	0.41	0.43	0.34	0.11	0.10	0.21

PKC, palm kernel cake; DC, decanter cake; CD, control diet; DCD, decanter cake diet; PKCD, palm kernel cake diet; CPOD, control plus 5% palm oil diet.

Rumen fluid samples were collected from each goat on the day before and on the day of the experiment, and pooled to give one sample per goat as time 0 before the introduction of the experimental diets. Additional rumen fluid samples were collected on days 4, 6, 8, 12, 18, 24, and 30 post-experimental diets. All samples were collected 2 h after the morning feeding, squeezed through four layers of cheese cloth and kept frozen (−20°C).

### Feed analysis

The proximate analysis of the experimental feed was performed following the standard methods of the Association of Official Analytical Chemists (AOAC) [17]. The DM was determined by oven drying in an air forced oven for 24 h at 105°C. Crude protein was determined using Kejltec Auto Analyzer after digestion in 72% H_2_SO_4_. Ether extract was determined in petroleum ether using Soxtec Auto Analyzer. The ash content was determined by ashing the feed samples in a muffle furnace at 550°C for 5 h. The samples were also analyzed for neutral detergent fiber (NDF) without sodium sulphite or heat stable amylase, and acid detergent fiber (ADF) according to Van Soest et al [18]. The results on NDF and ADF include residual ash.

Total fatty acids of feed samples were extracted following the method of Folch et al [19] modified by Rajion et al [20] using chloroform/methanol 2:1 (v/v). Methylation of fatty acids to their fatty acid methyl esters (FAME) was performed using KOH in methanol and 14% methanolic boron trifluoride (BF3). The FAME was separated by gas chromatography (Agilent 6890) equipped with injector and a flame ionization detector using capillary column of 100×0.25 mm×0.2 µm film thickness. The carrier gas was nitrogen at a flow rate of 1.2 ml/min. The injector temperature was programmed at 250°C, and the detector temperature was 270°C. The column temperature started to run at 150°C for 2 min, increased to 158°C at 1°C/min, held for 28 min, increased to 220°C at 1°C/min, and then held for 20 min.

### Rumen microbial DNA extraction

Total bacterial DNA was extracted using the QIAamp® DNA mini stool kit (Qiagen, Hilden, GmbH) according to manufacturer's protocol with a few modifications. Briefly, 1.4 ml of buffer ASL was added to 500 µl of rumen sample and was vortexed for 15 s. Following vortexing the sample was incubated at 95°C for 10 min. The sample was then vortexed again for 15 s and was centrifuged at 15,000×g for 1 min to pellet the cell debris and other suspended particles present in the sample. The supernatant was aspirated to a fresh 2 ml tube and an inhibitorEX tablet was added and vortexed immediately until the tablet was completely dissolved (∼1 min). The sample was then incubated at room temperature for 1 min to bind all inhibitors, and was centrifuged at 15,000×g for 3 min to remove the inhibitorEX bound other inhibitors. The solution was aspirated to a new 1.5 ml microcentrifuge tube and centrifuged for an additional 3 min to remove any residual inhibitor. Then 15 µl of proteinase K and 200 µl of AL buffer was added to 200 µl of the supernatant, vortexed and incubated at 70°C for 30 min. Then, 200 µl of 100% ethanol was added to the proteinase digested sample and the combined sample was applied to the QIAamp spin column and centrifuged at 15,000×g for 1 min to bind the DNA. The column was washed with 500 µl of buffer AW1 and then AW2 respectively. The spin columns were centrifuged at 15,000×g to remove residual buffer AW2 and the DNA was eluted using 200 µl of buffer AE. The concentration of the extracted DNA was measured using a UV spectrophotometer, and the quality of the DNA was evaluated on a 1.2% agarose gel.

### Real-time PCR

Species-specific real-time PCR was performed using Bio-Rad iCycler MyiQ single color real-time PCR detection system (Bio-Rad laboratories, INC), using fluorescence detection of SBYR Green mix. The SBYR Green mix, bacteria-specific primers, sample DNA template and RNAase free water were loaded into each well of PCR plate (Bio-Rad). The primers selected are presented in **Table 2** **.** Briefly, 5 µL SBYR Green mix, 1 µL of each primer, 2 µL of sample DNA template and RNAase free water were added to make a total volume of 10 µL. Amplification involved one cycle at 95°C for 10 min for initial denaturation and then 40 cycles at 95°C for 30 sec followed by annealing at 60°C for 30 sec, and then at 72°C for 1 min for general bacteria and *F. succinogesnes*. Amplification conditions for *R. albus* and *R. flavefaciens* were carried out similarly, except for annealing temperature of 55°C. Conditions for the methanogenic archaea were as follows: 30 sec at 94°C for denaturing, 30 s at 58°C for annealing and 90 s at 72°C for extension as reported by Wright et al [21]. Negative controls (without DNA template) were run with every assay to assess overall specificity. Detection of the fluorescent product was set at the last step of each cycle. To determine the specificity of amplification, a melting curve was obtained by slow heating with a 0.1°C/sec increment from 65°C to 95°C, with fluorescence collection at 0.1°C intervals. Instead of amplifying the target genes from individual community DNA samples and then pooling the PCR products, a sample derived standard was prepared from the treatment pool set of community DNA, and then the QIAquik PCR purification kit was used to purify the PCR products and quantified using spectrophotometry. For each sample derived standard, the 16 S rRNA gene copy numbers were calculated based on the length of PCR product and the mass concentration [22]. Samples were performed in duplicate along with standards of known bacterial DNA concentrations, which were prepared in 10-fold dilutions. Samples and known standards (10^−1^ to 10^−8^ ng) were assayed on the same plate to allow for the quantification of bacteria present in the sample.

**Table 2:** PCR primers for real time-PCR assay.

Target species	Forward/reverse	Primer sequence (5′-3′)	Annealing temperature	Amplicon (pb)	Reference
Total bacteria	F	CGG CAA CGA GCG CAA CCC	60°C	132	14
	R	CCA TTG TAG CAC GTG TAG CC			
R. albus‡	F	CCCTAAAAGCAGTCTTAGTTGG	55°C	175	33
	R	CCTCCTTGCGGTTAGAACA			
R. flavefacien‡	F	CGAACGGAGATAATTTGAGTTTACTTAGG	60°C	130	33
	R	CGGTCTCTGTATGTTATGAGGTATTACC			
F. succinogenes	F	GTTCGGAATTACTGGGCGTAAA	55°C	121	33
	R	CGCCTGCCCCTGAACTATC			
Methnogenic archea	F	TTCGGTGGATCDCARAGRGC	58°C	140	45
		GBARG TCGWA WCCGT AGAAT CC			

For each standard, linear regression derived from the threshold cycle (C_T_) of each DNA dilution versus the log quantity was calculated. Logarithm of DNA concentrations (copies/ml) was plotted against the C_T_ means obtaining a straight line of the equation:

y  =  mx + b, where y  =  C_T_ value, m  =  slope, and x  =  log quantity.

From the equation for the linear regression we can derive the following equation to determine the quantity N in unknown samples: N_n_  =  10^(n – b/m)^, where n  =  C_T_.

### Statistical analysis

Data were analyzed as repeated measurements in time by using Mixed procedure of SAS [23]. The model used was: Y_ijk_  =  μ + D_i_ + A_j_ + T_k_ + (DT)_ik_ + e_ijk_, where Y_ijk_  =  values of observations, μ  =  general mean, D_i_  =  effect of the day of sampling, A_j_  =  effect of animal, T_k_  =  effect of dietary treatment and e_ijk_  =  residual error. The Duncan multiple range test was used to further compare means at P<0.05.

## Results and Discussion

Palm oil extraction produces various biomass including fiber, shell, empty fruit bunch, DC and PKC. The latter is originated from the kernel and being used as a ruminant feed. The DC is a by-product obtained after dehydration of palm oil mill effluent and is characterized by considerable variability in chemical composition.

**Figure 1:**
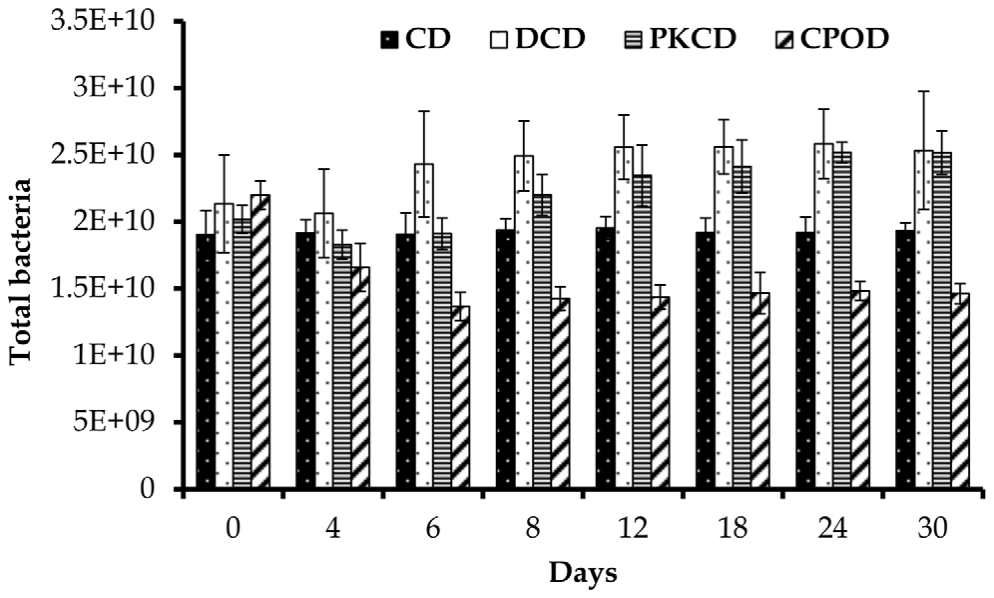
Effect of dietary treatments on population of total bacteria (×10^10^/ml) in the rumen of goats at different days of feeding. CD (control diet), DCD (decanter cake diet), PKCD (palm kernel cake diet) and CPOD (control+5% palm oil diet). Vertical bars are standard error of the mean (SEM).

The effect of dietary treatments on total bacteria, methanogenic archaea, and the three cellulolytic bacteria are presented in **Table 3** **.** Total bacteria were found to be significantly (P<0.01) higher in goats fed DCD and PKCD than those fed CD and CPOD. Moreover, the copy numbers of total bacteria were significantly (P<0.01) affected by day of sampling, but not by diet × day of sampling interaction (**Figure 1**). Previous reports indicated that the dietary lipid supplementation could inhibit the growth of bacteria in the rumen of cattle fed soy bean oil or linseed oil [24], [25], but the dietary oil addition had no effect on the total bacteria numbers in sheep [26]. More recently it was suggested that determination of growth of bacteria in response to the dietary oil supplement, especially unsaturated oil, should be made by examining specific bacteria species instead of the density of total bacteria [27]. The total bacteria concentration in rumen fluid of goats fed PKCD and DCD was higher (P<0.05) than those in goats fed the CD and CPOD. Moreover, in the present study protozoa numbers in the rumen decreased rapidly to a very low concentration 4 days after feeding CPOD, DCD and PKCD, and totally disappeared from the rumen of goats fed the CPOD on day 24, and in goats fed both the DCD and PKCD on day 30 [16]. It is well established that oils are toxic to rumen ciliate protozoa [28], [29], mainly due to their limited ability to absorb and transform lipids, resulting in swelling and consequent rupture of the protozoa cells [30]. In the present study, ether extract for the diet CD was almost 3-fold lower than in the other diets (DCD, PKCD and CPOD) indicating that the concentration of lipids in the CD diet was not enough to affect the concentration of rumen protozoa. It is known that such a reduction [31] and long or short term defaunation [32] may increase the rumen bacterial population. Therefore, the increased population of total bacteria in the goats fed DCD and PKCD could be due to multiplication of bacteria after the elimination of protozoa that are known to engulf rumen bacteria. However, the case was not similar in the goats fed CPOD where both rumen ciliate protozoa and total bacteria were decreased probably due to the direct inclusion of PO in the diet.

**Table 3:** Table **3.** Effect of dietary treatments and day of sampling on microbial population (copies/ml) in the rumen of goats.

Item	Diet^1^					*P*-value		
Species	CD	DCD	PKCD	CPOD	SEM	Diet	Day	Diet × day
Total bacteria (×10^10^)	1.9^b^	2.4^a^	2.2^a^	1.7^c^	0.7	**	**	NS
*F. succinogenes* (×10^9^)	5.0^a^	5.7^a^	5.1^a^	3.5^b^	0.9	*	*	NS
*R. albus (×10^6^)*	7.4^b^	13.6^a^	16.8^a^	5.5^b^	0.4	**	**	**
*R. flavefaciens* (×10^7^)	4.6^ab^	5.2^a^	4.9^a^	4.3^b^	0.3	**	NS	NS
Methanogenic archea (×10^9^)	2.48^a^	1.96^ab^	1.50^b^	1.32^b^	0.02	**	***	*

1 CD (control diet), DCD (decanter cake diet), PKCD (palm kernel cake diet) and CPOD (control+5% palm oil diet).

NS not significant statistically (*P*>0.05).

a,b,c Means in the same row with different superscripts are significantly different.

*P<0.05, **P<0.01, ***P<0.001.

**Figure 2:**
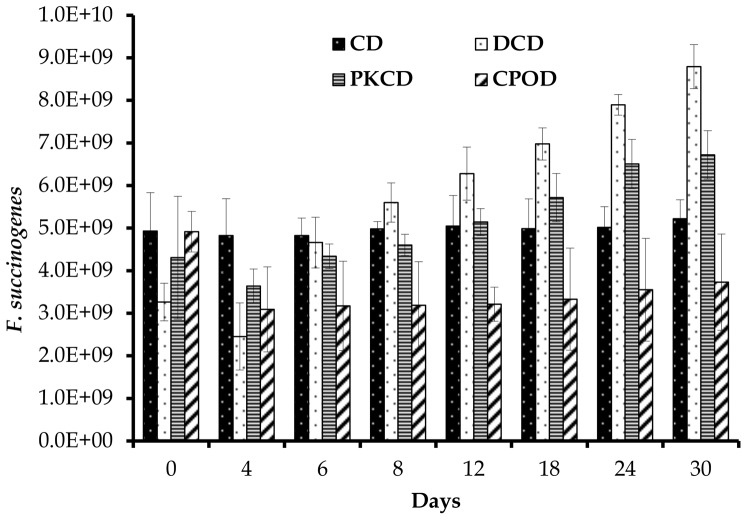
Effect of dietary treatments on population of *F. succinogenes* (×10^9^/ml) in the rumen of goats at different days of feeding. CD (control diet), DCD (decanter cake diet), PKCD (palm kernel cake diet) and CPOD (control+5% palm oil diet). Vertical bars are standard error of the mean (SEM).

**Figure 3:**
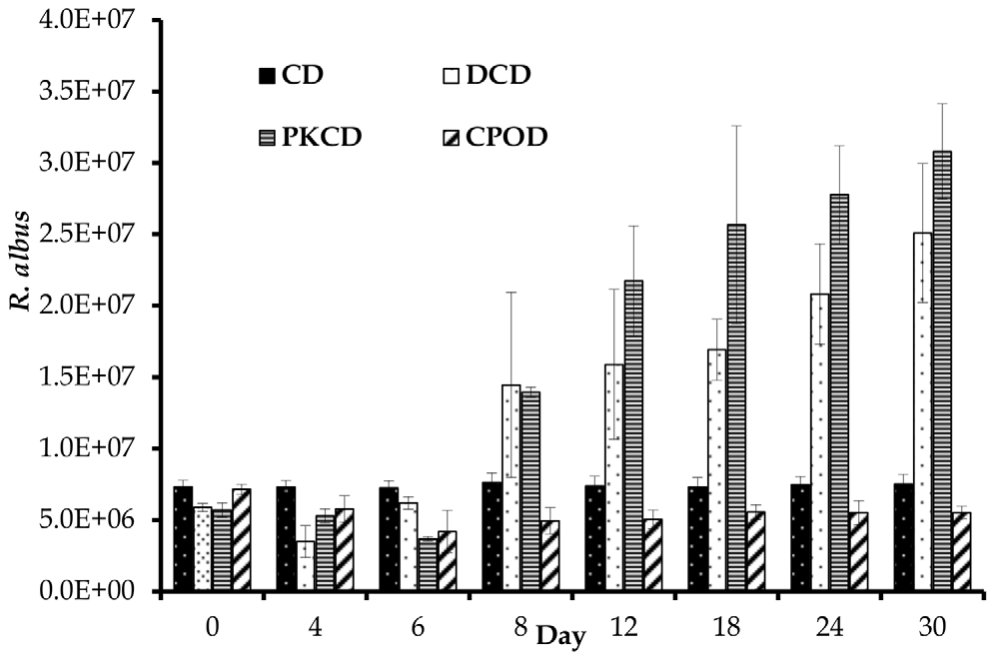
Effect of dietary treatment on population of *R. albus* (×10^6^/ml) in the runm of goats at different days of feeding. CD (control diet), DCD (decanter cake diet), PKCD (palm kernel cake diet) and CPOD (control+5% palm oil diet). Vertical bars are standard error of the mean (SEM).

**Figure 4:**
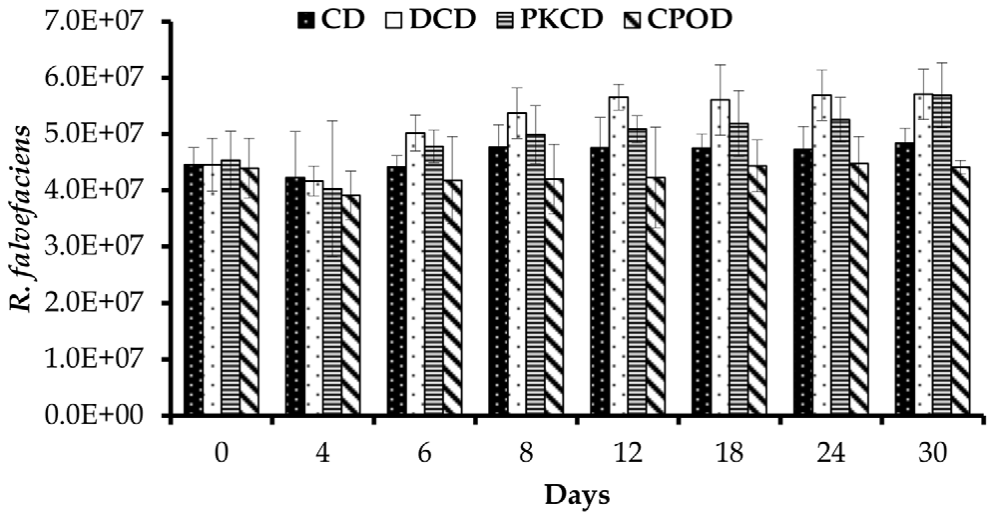
Effect of dietary treatments on population of *R. flavefaciens* (10^7^/ml) in the rumen of goats at different days of feeding. CD (control diet), DCD (decanter cake diet), PKCD (palm kernel cake diet) and CPOD (control+5% palm oil diet). Vertical bars are standard error of the mean (SEM).

In the present study, the copy number of *F. succinogenes* was highest among the three cellulolytic bacteria, followed by *R. flavefaciens* and then *R. albus*. Similar trends were observed in swamp buffalo [4] and sheep [33]. Additionally, the population size of *F. succinogenes* was significantly (P<0.05) reduced in goats fed CPOD compared to those fed the other dietary treatments. Moreover, this bacterium showed a decreased trend between day 0 and 4 for goats fed DCD, PKCD and CPOD, and then started to increase during the rest of the experimental period in the goats fed DCD and PKCD (**Figure 2**). However, the population size of *F. succinogenes* stayed lower in the group fed CPOD and stable in CD group during the entire period of the experiment. Goats fed DCD and PKCD had significantly higher (P<0.01) population of *R. albus*, additionally, this bacteria was significantly affected (P<0.01) by day of sampling and diet × day of sampling interaction. Similar to *F. succinogenes*, the concentration of *R. albus* was reduced between days 0 and 4 and increased between days 4 and 6 for the groups fed PKCD and CDC (**Figure 3**). The concentration of *R. flavefaciens* was significantly higher (P<0.01) in goats fed DCD and PKCD in comparison to those fed CD and CPOD. Like the other two species, *R. flavefaciens* population size was reduced between days 0 and 4 for goats fed DCD, PKCD and CPOD, and then a slight increase was observed for DCD and PKCD (**Figure 4**).

Diet is the most important measured factor that affects the microbial community structure in the rumen [25]. Therefore, the decrease in rumen microorganisms between day 0 and 4 might be related to the dietary shift from CD to experimental diets. Different dietary fatty acids were reported to have different effects on ruminal bacteria. The effects differ from inhibition to no effect according to fatty acid composition [34]–[37] and level of fatty acids included in the diet [25]. Moreover, most ruminal bacteria species were able to grow in the presence of 50 µg linoleic acid per ml of medium, but not at 200 µg per ml of medium [25]. In contrast, feeding 4% soybean oil and/or linseed oil inhibit the growth of cellulolytic bacteria in the rumen of dairy cattle [38]. In the present study, 5% PO resulted in decreased DNA copy numbers of total bacteria and cellulolytic bacteria studied. However, feeding DCD and PKCD resulted in increased copy numbers of the three representative cellulolytic bacteria in the rumen fluid of goats although the fatty acid profile of DC is almost similar to PO. This increase might be explained by inhibitory effect of the fatty acid present in the two diets on rumen protozoa or certain bacteria species that suppress the growth of cellulolytic bacteria. Many interactions among ruminal bacteria have been characterized before [39]–[40]. For example, Chen and Weimer [39] reported that the growth of *R*. *flavefaciens* and *F*. *succinogenes* were suppressed by inhibitors produced by *R*. *albus*. Therefore, one may expect that the increase in DNA copy number of *R. albus* and *R. flavefaciens* in the DCD and PKCD fed goats may have resulted from the reduction in protozoal counts, or the DNA copy number of other bacteria species.

**Figure 5:**
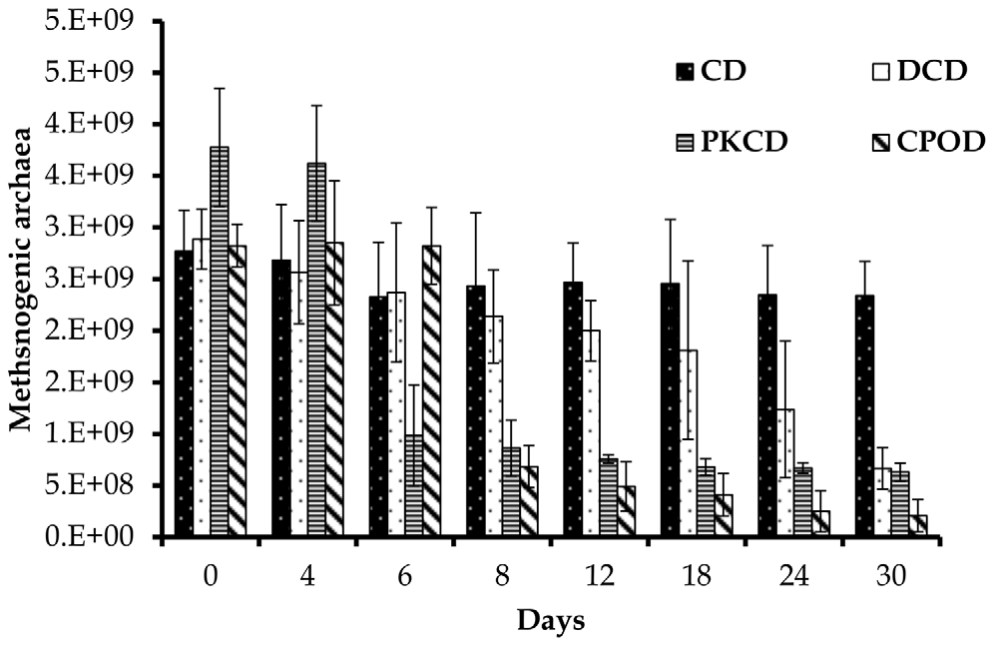
Effect of dietary treatment on population of total methanogenic archaea (×10^9^/ml) in the rumen of goats at different days of feeding. CD (control diet), DCD (decanter cake diet), PKCD (palm kernel cake diet) and CPOD (control+5% palm oil diet). Vertical bars are standard error of the mean (SEM).

In the present study, feeding PKCD and CPOD significantly reduced (P<0.001) the density of the methanogens in the rumen of goats, whilst the DCD fed goats had no effect in contrast to the CD fed goats. Moreover, the reduction in methanogen numbers was found to be affected by day of sampling (P<0.001) and diet × day of sampling interaction (P<0.05). Reduction of methanogens was rapid between days 4 and 6 in the goats fed PKCD and CPOD respectively, whereas a constant trend was observed in the CD fed goats (**Figure 5**). Methane originated during the process of fermentation in the rumen is an energy loss to the ruminant host and contributes to emission of greenhouse gasses into the environment [41]. Oils, especially those containing medium chain fatty acids are known to have the potential to suppress rumen methanogenesis and methanogens due to their toxic effects. Several studies reported that coconut oil, which contains about 21% myristic acid, consistently reduced rumen methanogenesis [42]–[45]. Fatty acid profile of PKCD is dominated by medium chain fatty acids (lauric and myristic) derived from PKC, and this may explain the decrease in the density of methanogens in the present study.

### Conclusion

The by-products PKC and DC could be an alternative low cost feed resource available for ruminants in the tropical areas where most of feed ingredients are imported. Reduced numbers of protozoa and methanogens in the rumen of goats fed PKCD and DCD may be associated with reduced methane production. Cellulolytic bacteria tested in the present study showed increased population in the rumen of the goats fed PKCD and DCD. Nevertheless, more research is required to evaluate whether this increase could reflect in an improved production characteristics.
